# Supplementary figures and images for: Modelling the impact of non-pharmaceutical interventions on workplace transmission of SARS-CoV-2 in the home-delivery sector
Source: PLoS One. 2023 May 5;18(5):e0284805. doi: 10.1371/journal.pone.0284805 (PMC10162531; doi:10.1371/journal.pone.0284805)

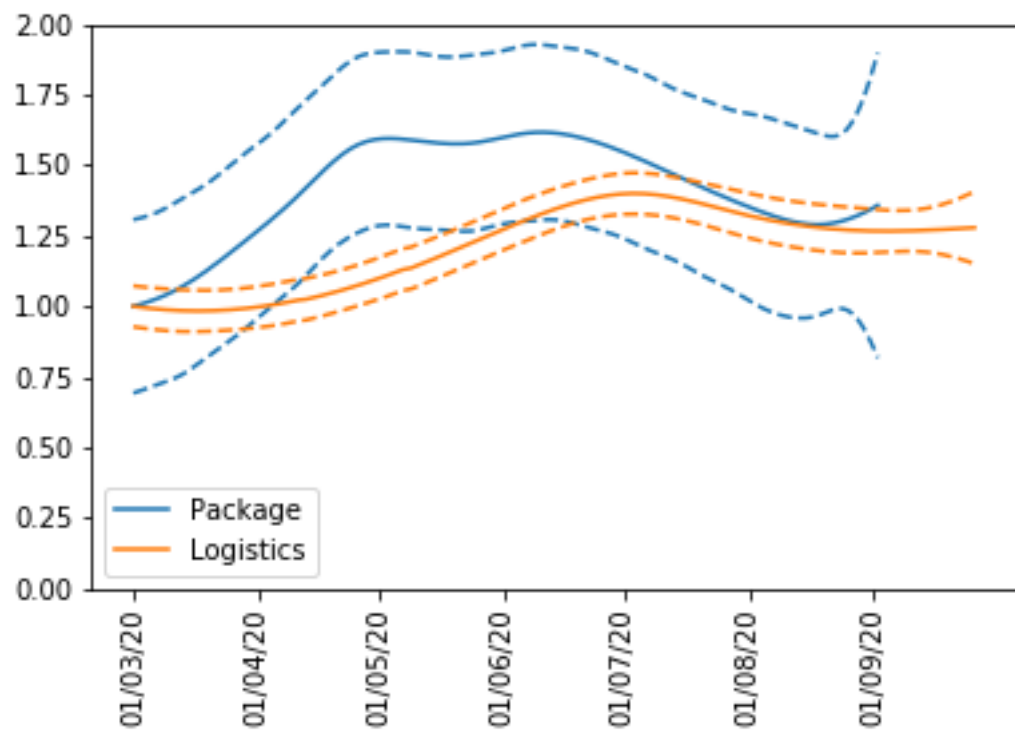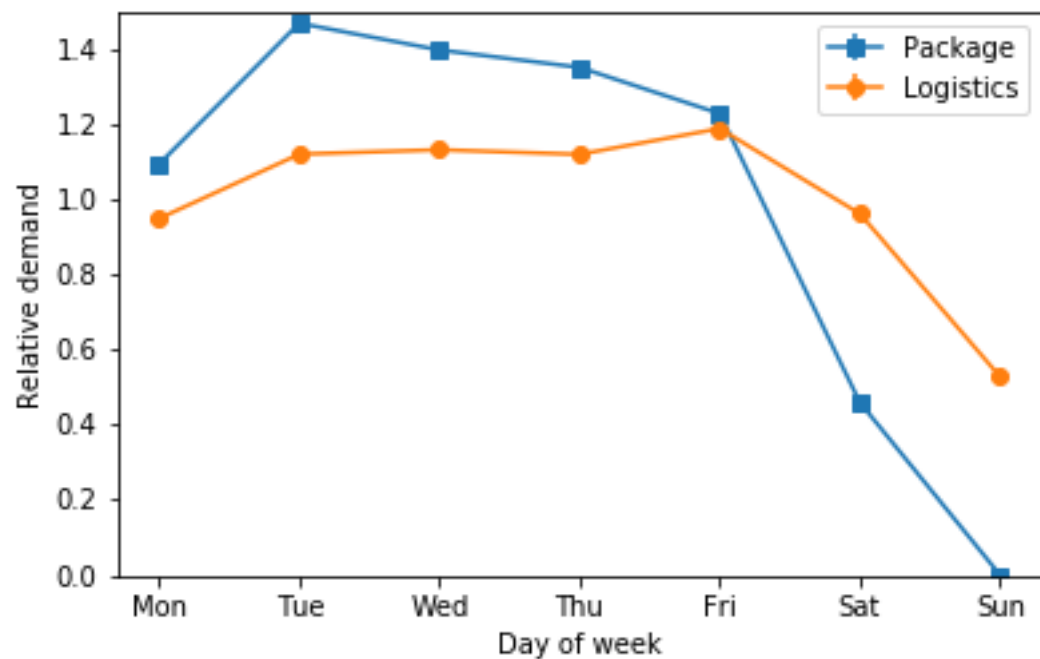

Supplement: S1 Fig — (a) Smoothed demand curves, fitted using a linear GAM, to company-wide figures for number of consignments from the parcel and logistics companies. The figures are given relative to their value at 01/03/20. (b) Weekday dependence for number of drivers and deliveries fitted using negative binomial regression. Each point shows the number of deliveries or drivers relative to the number on a Friday. (PDF) [file pone.0284805.s005.pdf]

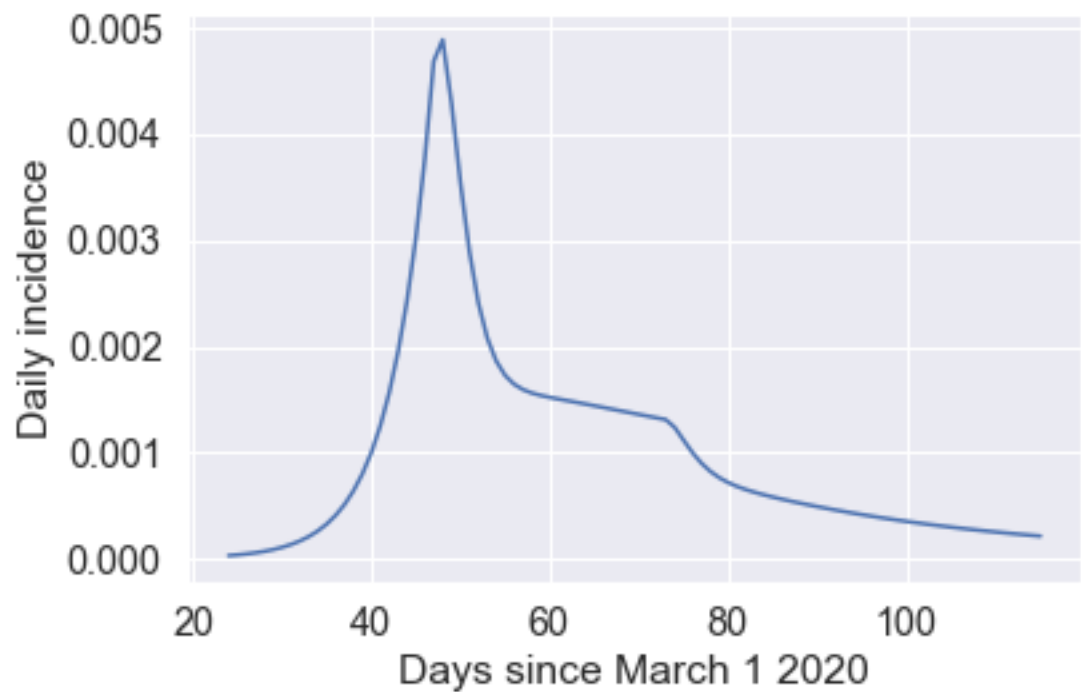

Supplement: S2 Fig — Community incidence rates assumed for the 3-month period simulated in the continuous-source outbreak scenario. (PDF) [file pone.0284805.s006.pdf]

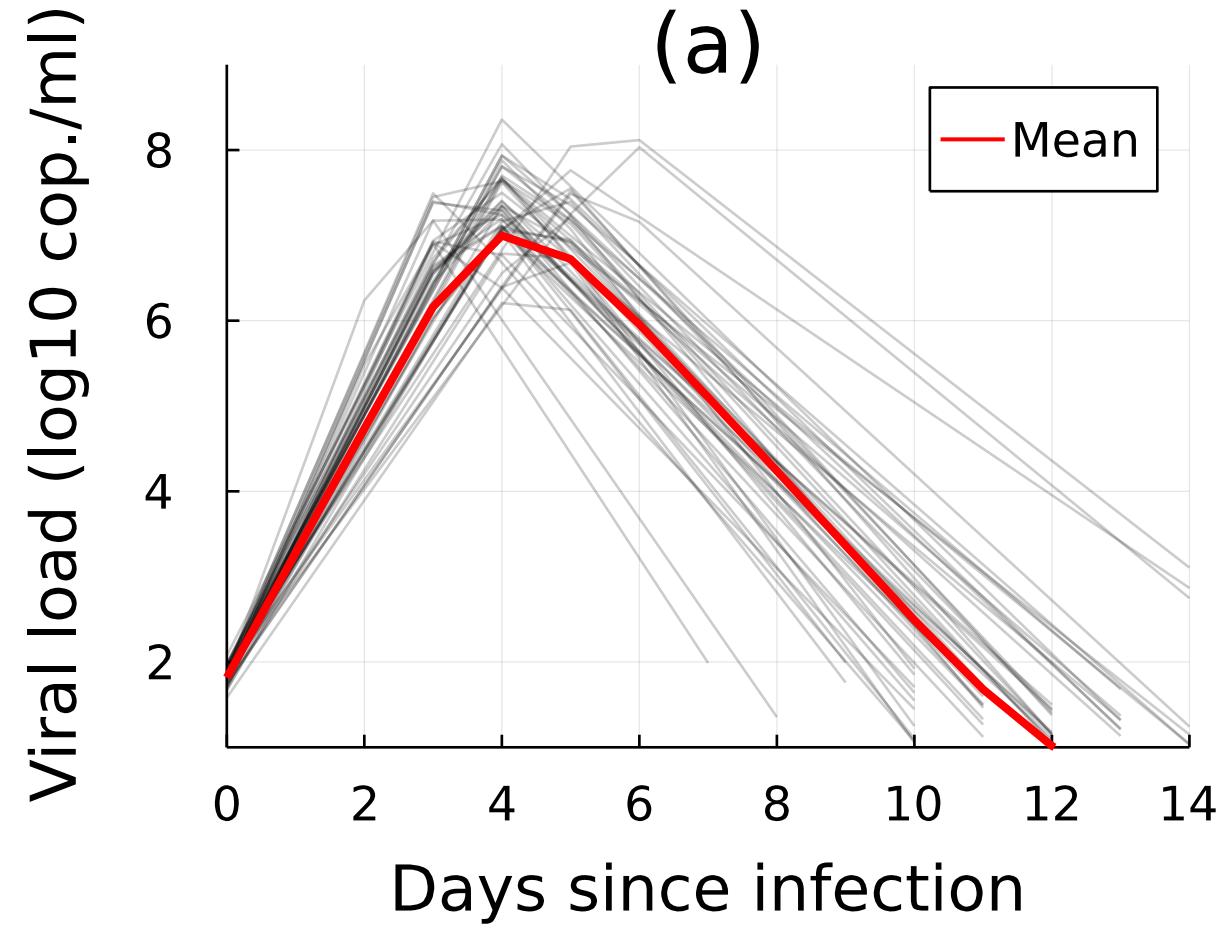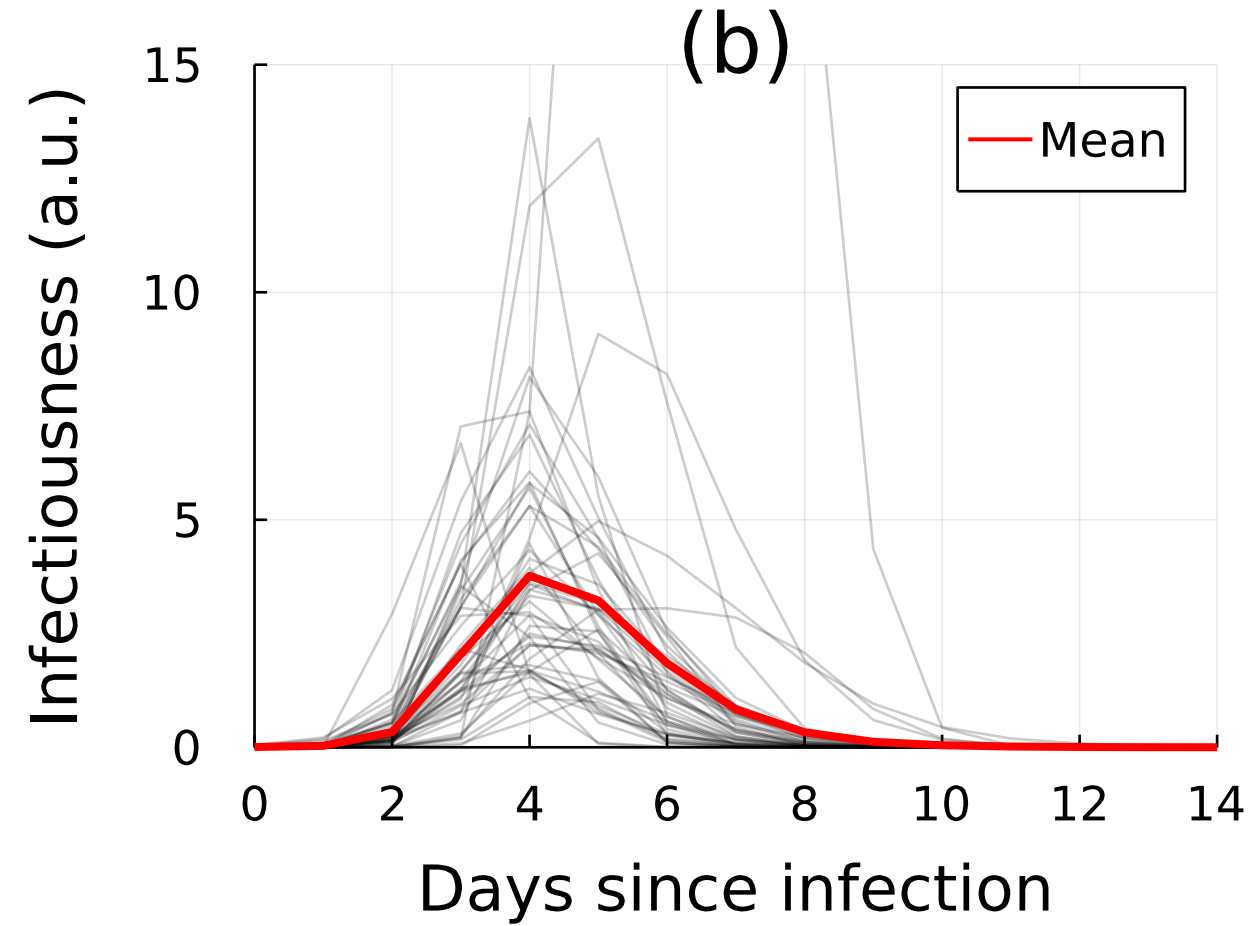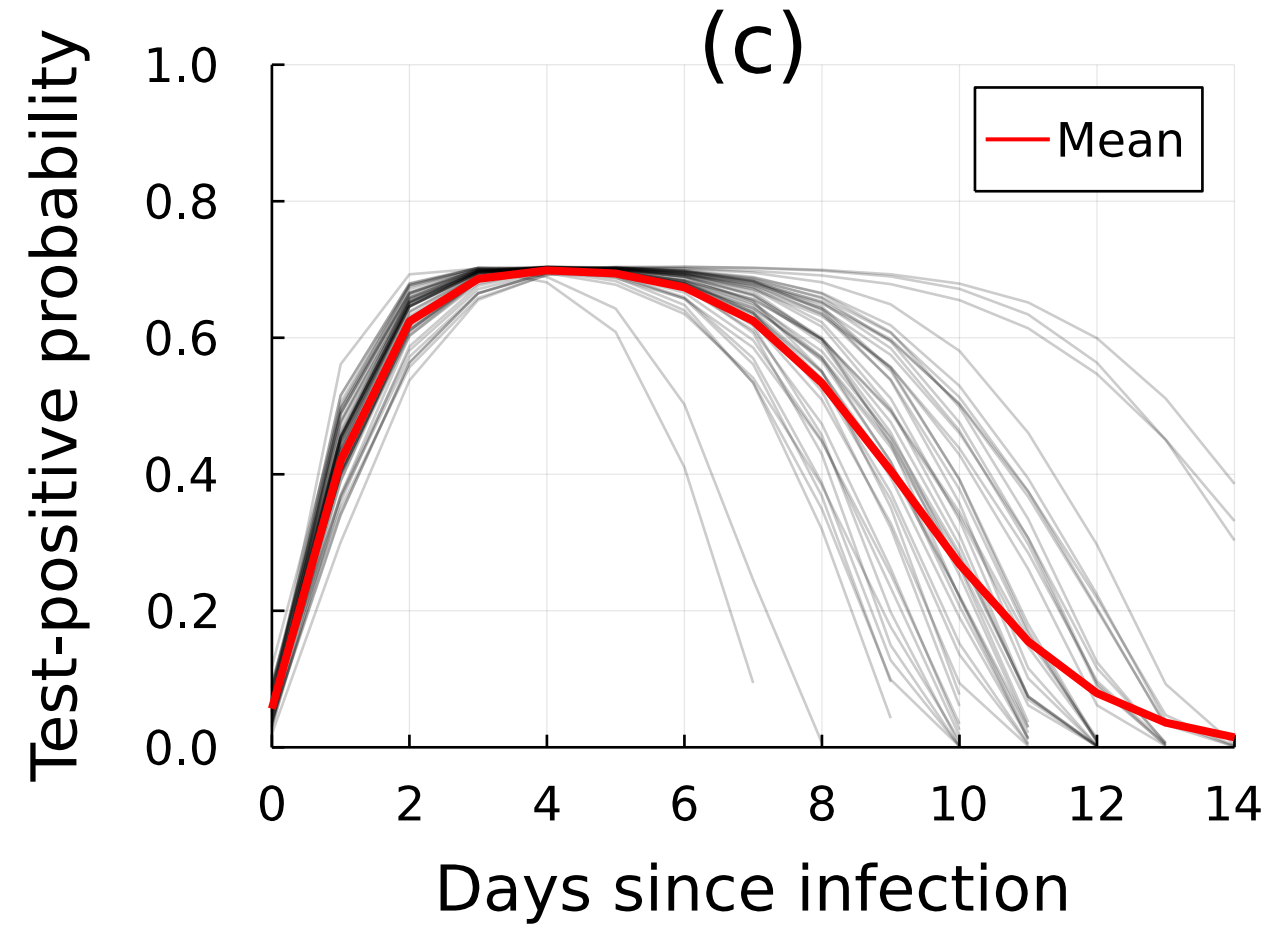

Supplement: S3 Fig — Each figure shows 50 randomly generated profiles of (a) RNA viral load (log10 copies/ml) and their associated (b) infectiousness (normalised units) and (c) test-positive probability. The red lines show the mean of 10,000 generated individuals at each time point (where a missing value is taken as 0). (PDF) [file pone.0284805.s007.pdf]

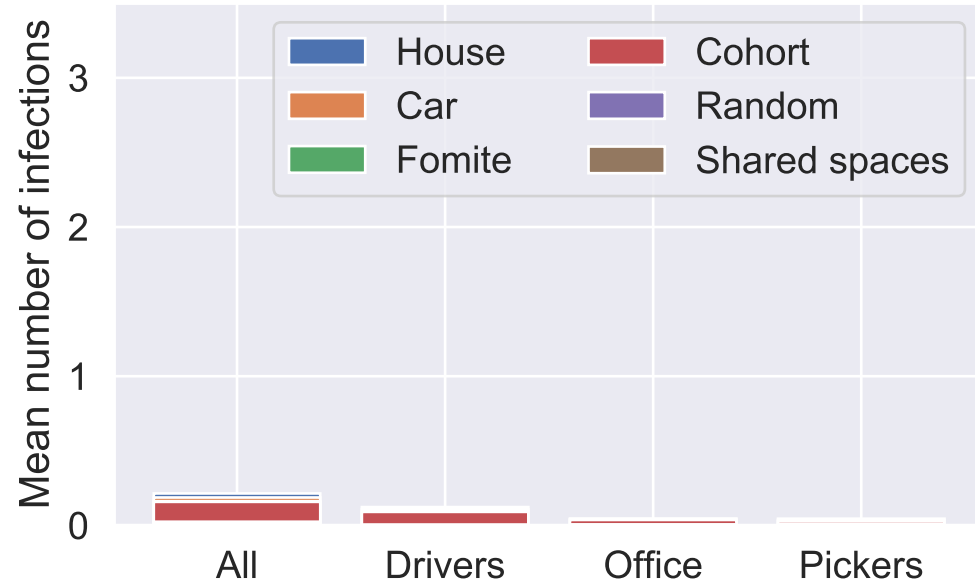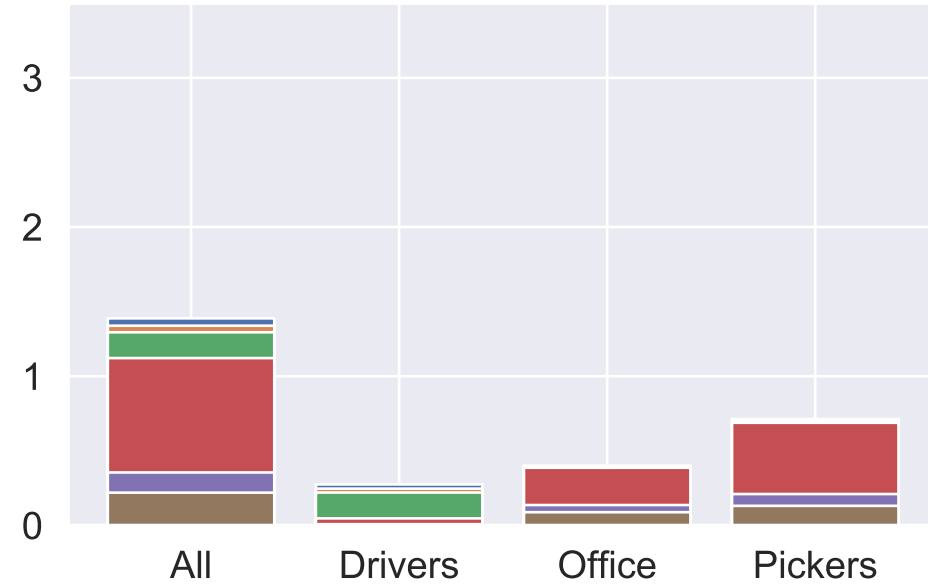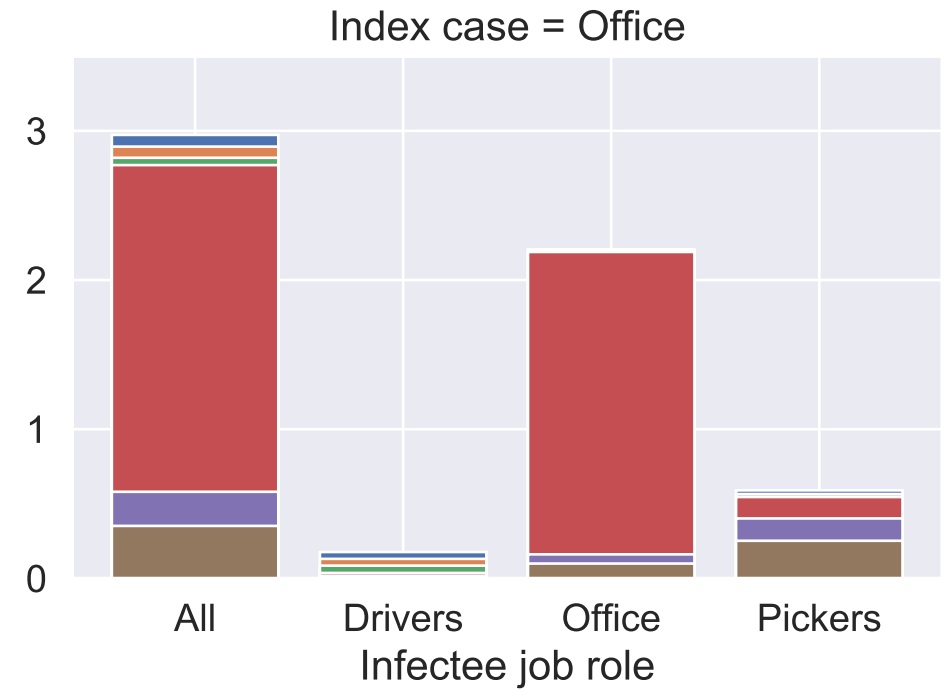

Supplement: S4 Fig — Stacked bar charts of the mean number of simulated secondary infections resulting from a single index case in (a) a driver, (b) a picker, or (c) an office worker in the SPDD work setting. Each bar shows secondary infections in each group of staff broken down by transmission route, as recorded in Table 2. Note that the “shared spaces” contacts does not include contacts from sharing an office, these are counted as “cohort” interactions for office staff. (PDF) [file pone.0284805.s008.pdf]

Random pairings

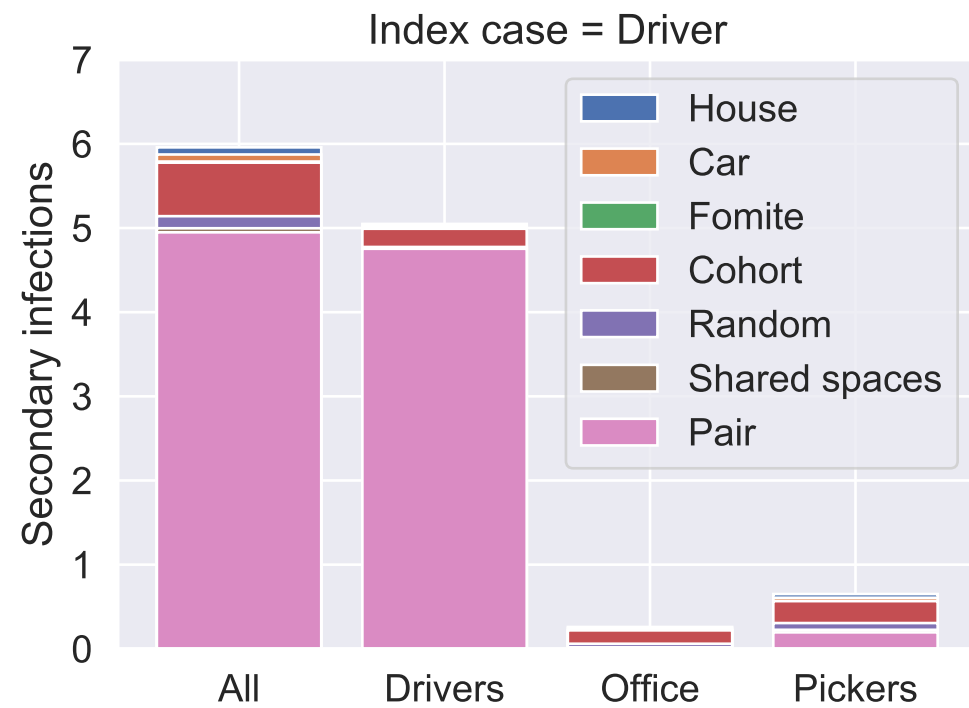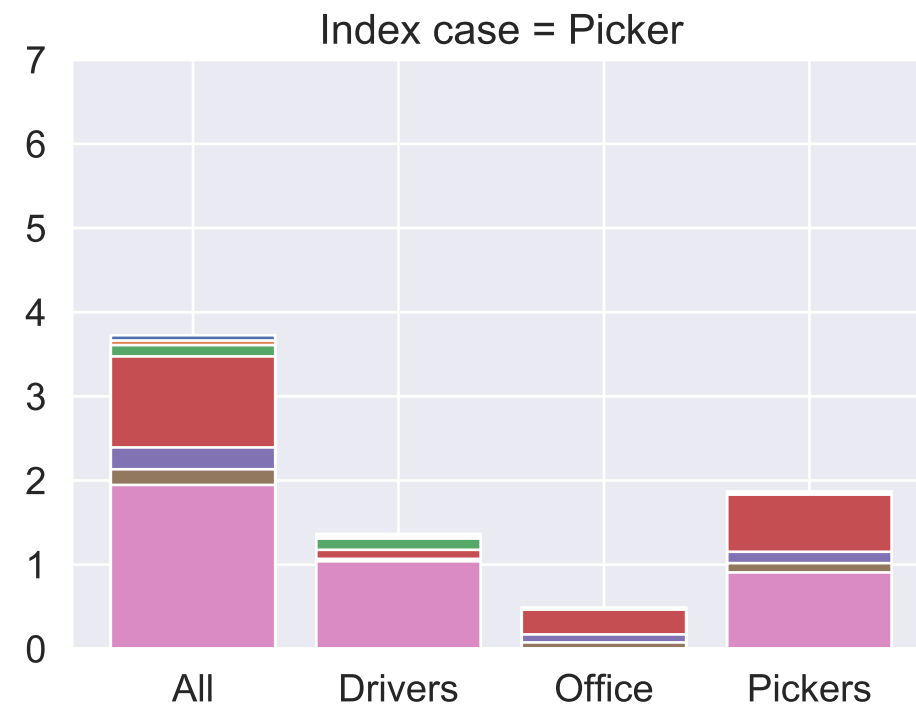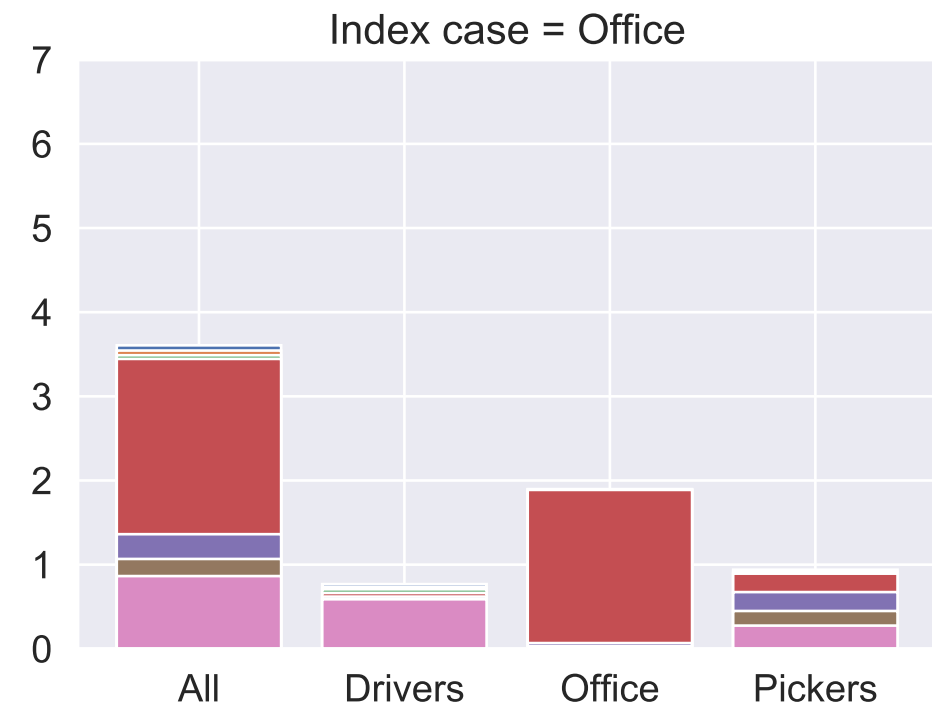

Fixed pairings

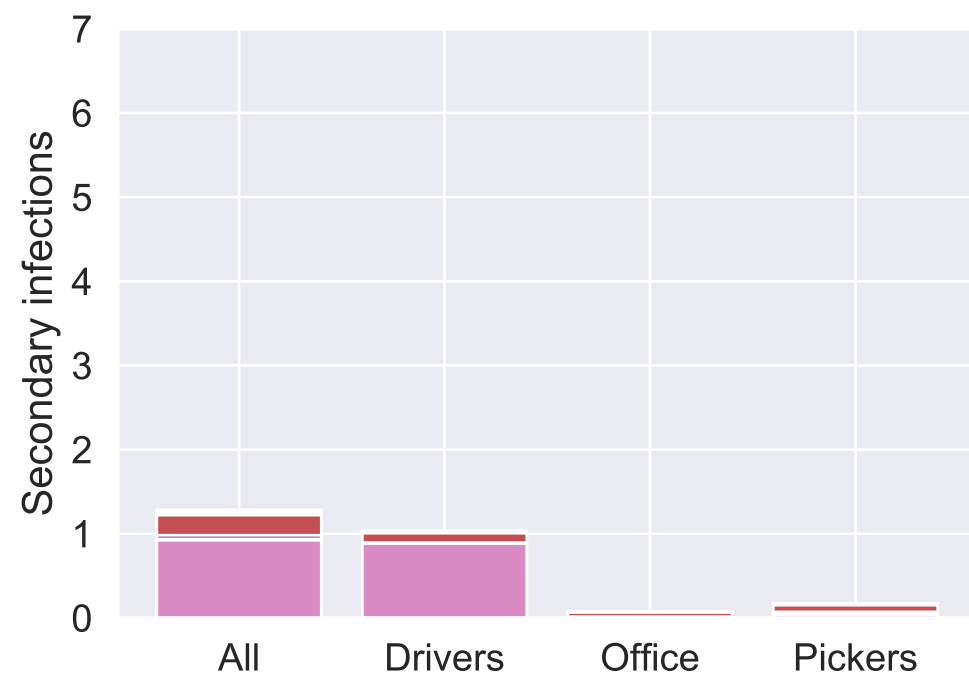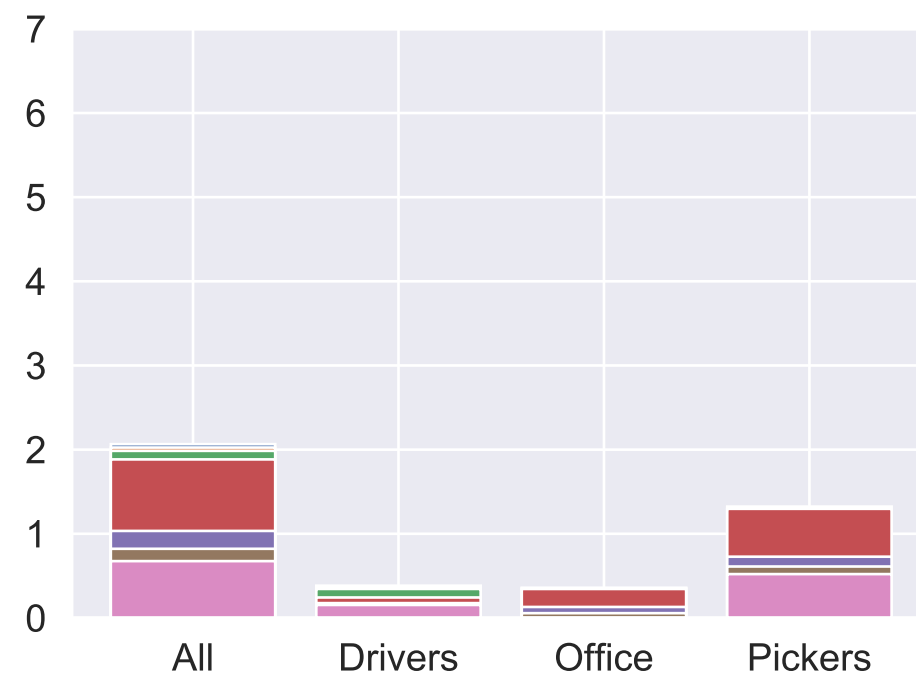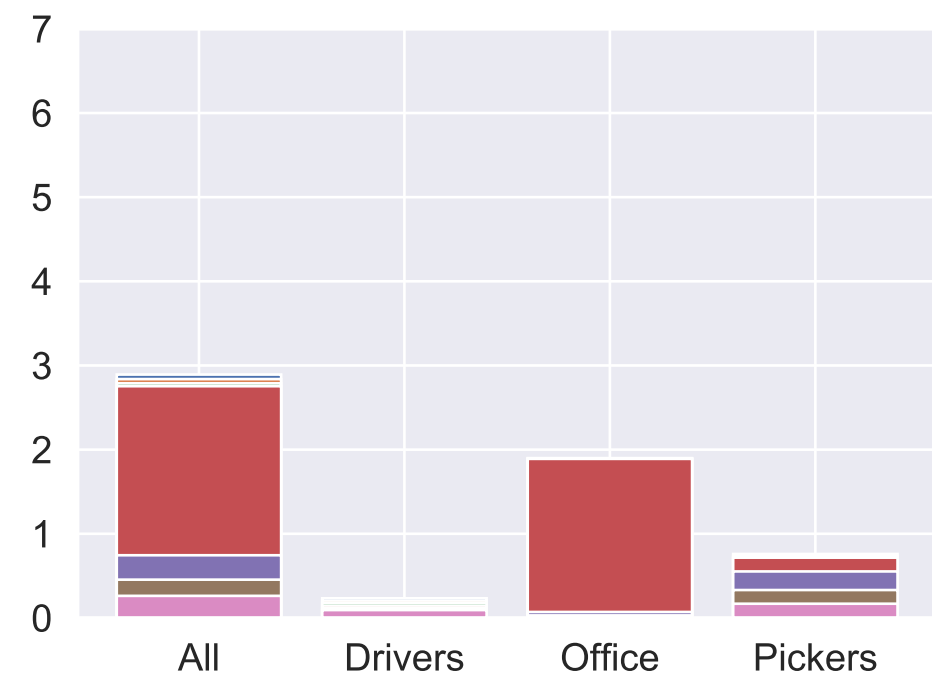

Supplement: S5 Fig — Stacked bar charts of the mean number of simulated secondary infections resulting from a single index case in (a) a driver, (b) a picker, or (c) an office worker in the LIDD setting. Each bar shows secondary infections in each group of staff broken down by transmission route, as recorded in Table 2. Note that the “shared spaces” contacts does not include contacts from sharing an office, these are counted as “cohort” interactions for office staff. (PDF) [file pone.0284805.s009.pdf]

(a) Index Case = Driver

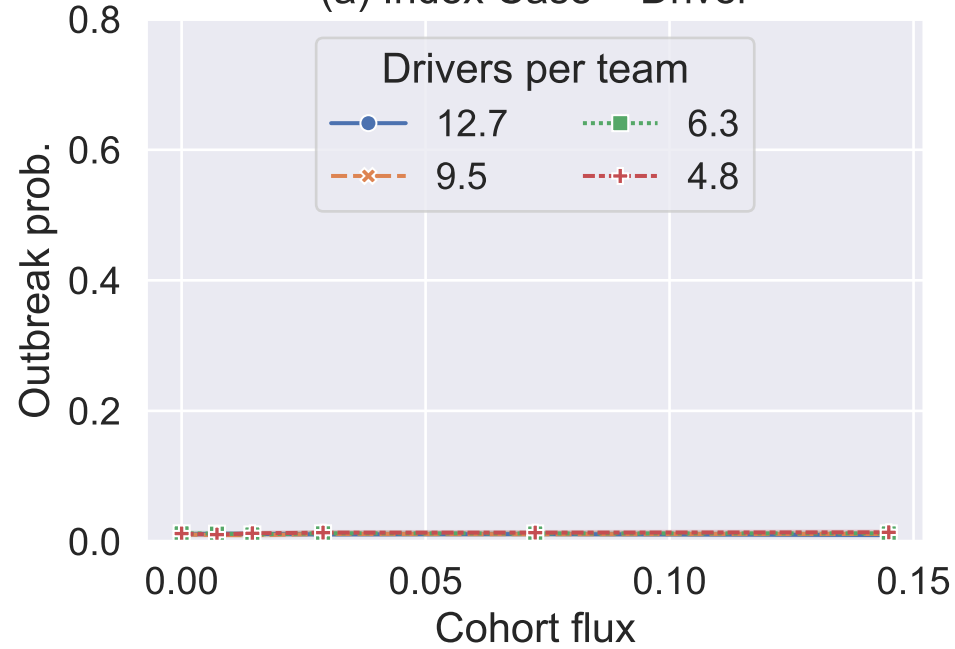

(b) Index Case = Picker

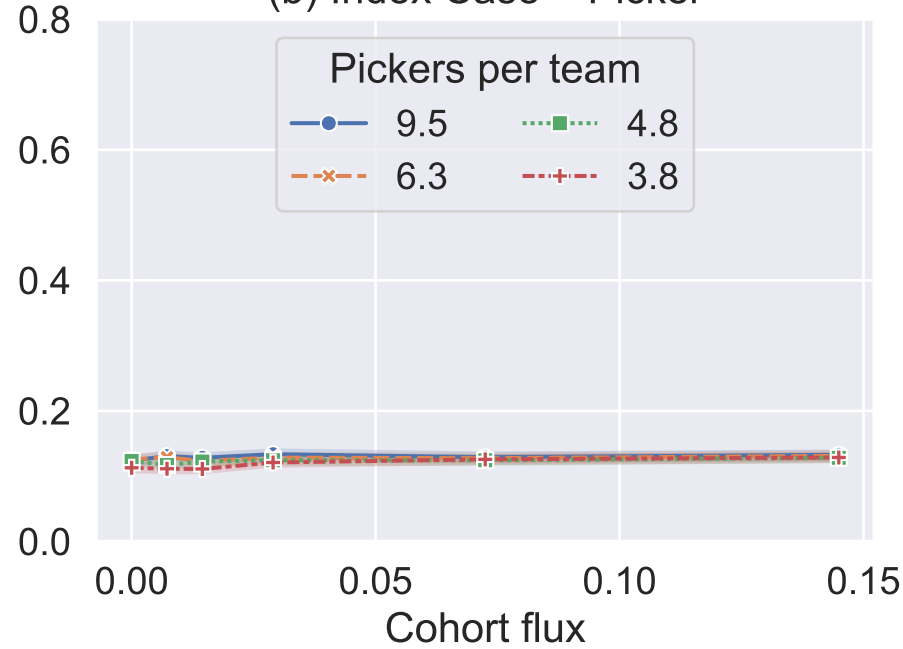

(c) Index Case = Office

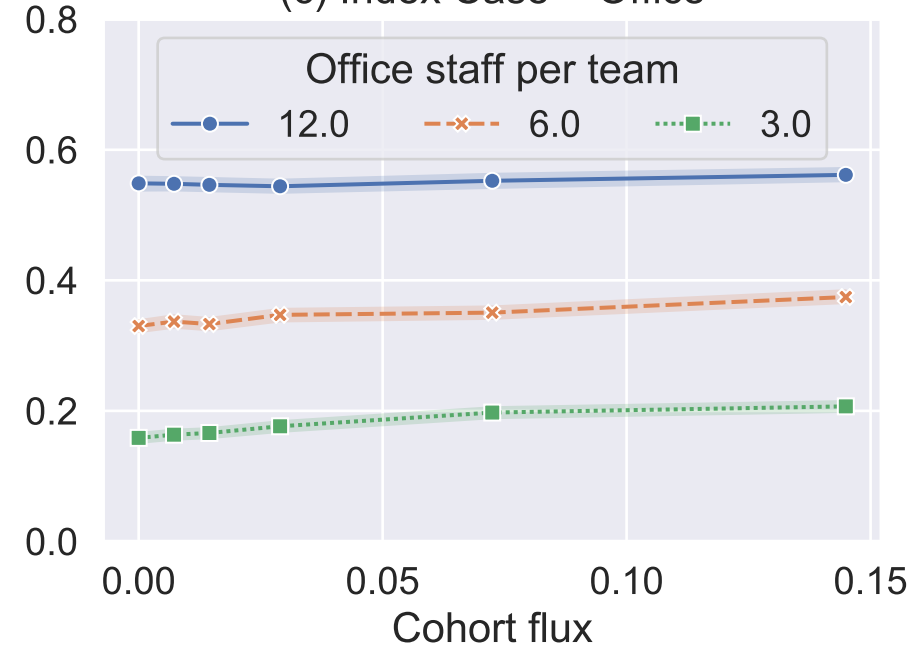

Supplement: S6 Fig — Estimated probability of outbreak (defined as more than 3 secondary cases) resulting from a single index case plotted against the cohort flux fc in days−1. Each marker shows the mean of 10,000 simulations, with shaded error region estimated using a bootstrapping process [38]. Point-source outbreaks where the source case was (a) a driver, (b) a picker; (c) an office worker. Each line in each figure compares simulations with different numbers of teams used for that job role, shown as the number of workers per team on average. In each figure, the job roles not shown have the default team size and pisol = 0.9 is assumed. (PDF) [file pone.0284805.s010.pdf]

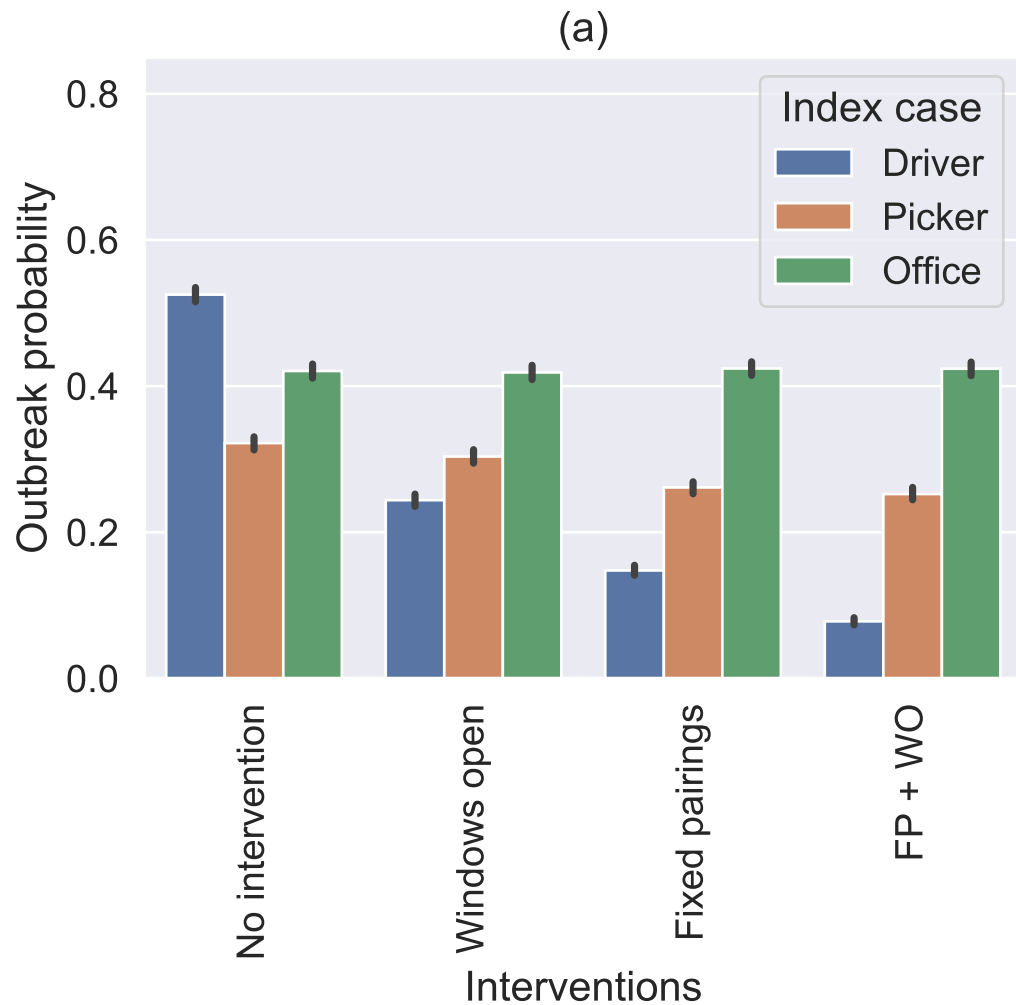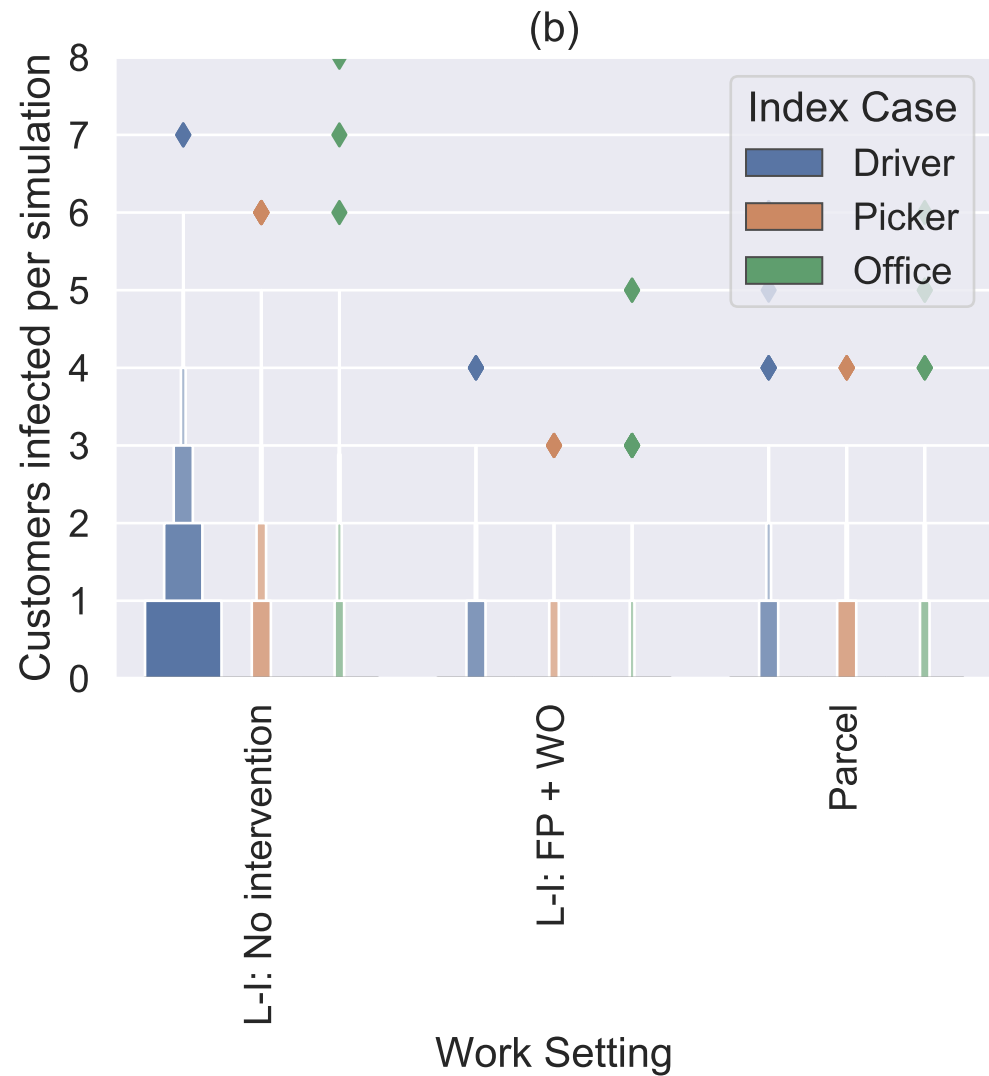

Supplement: S7 Fig — (a) Simulated probability of an outbreak (defined as more than 2 secondary cases). Four scenarios are shown: no intervention (staff are randomly paired each day); driver pairs travel with window open (transmission rate constant reduced to 1/5 of original value in this setting); fixed pairs (people always work with the same partner); and both of these interventions simultaneously (fixed pairs and windows open). Each bar represents 10,000 simulations, error bars indicate uncertainty in the mean, estimated via a bootstrapping method [38]. (b) Boxen plots of the number of customers infected per point-source outbreak simulation in the LIDD setting with either no or both interventions and the parcel delivery setting with default parameters. (PDF) [file pone.0284805.s011.pdf]

(a) Index Case = Driver

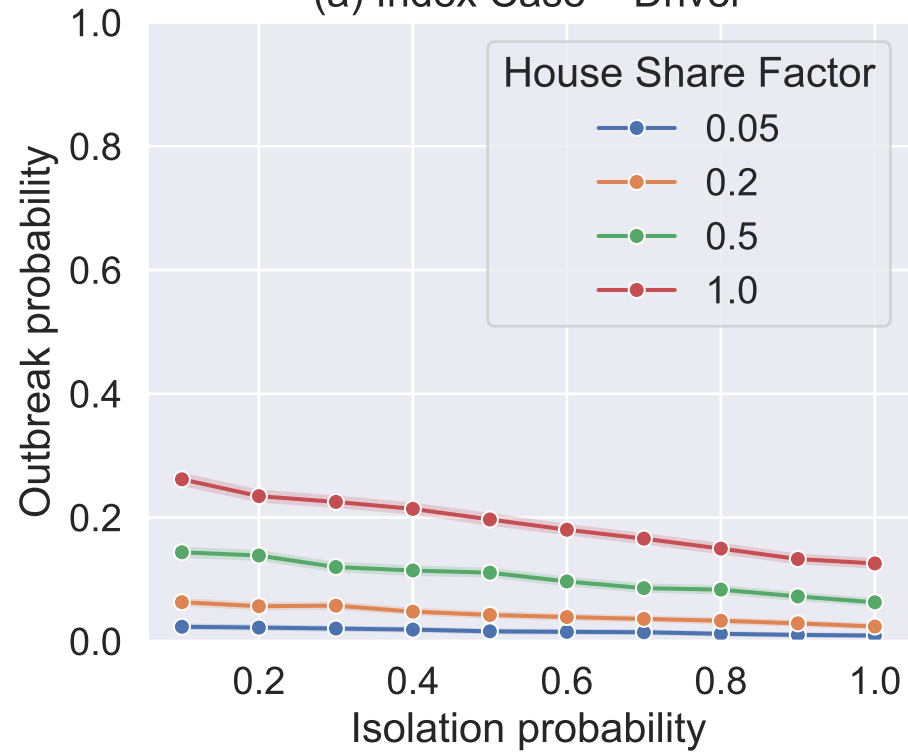

(b) Index Case = Picker

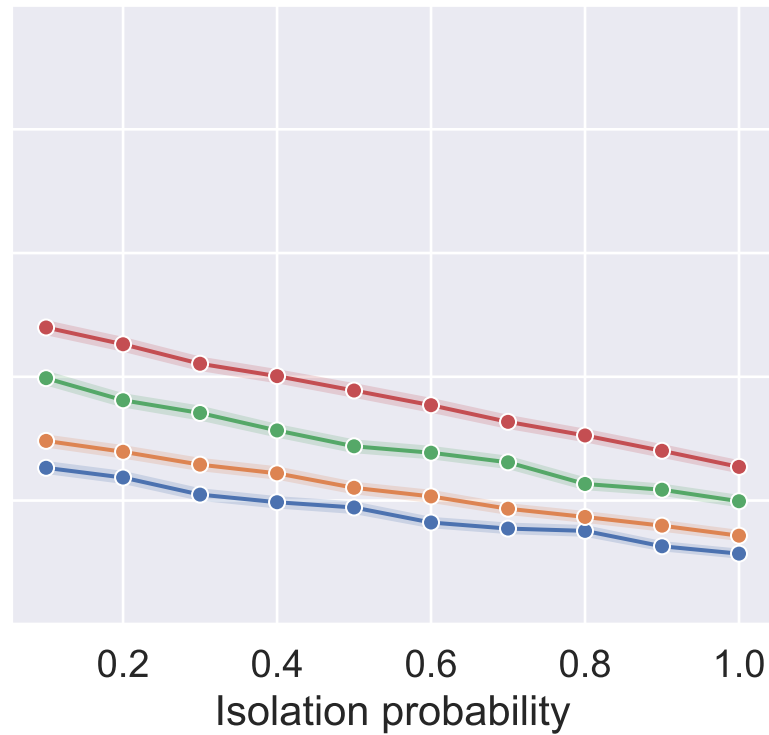

(c) Index Case = Office

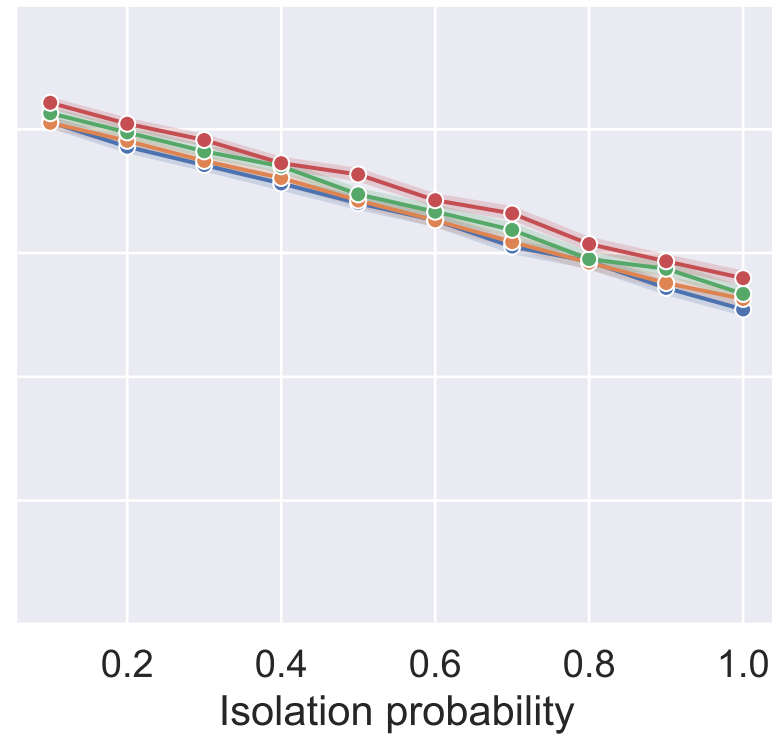

Supplement: S8 Fig — Dependence of simulated outbreak probability on the self-isolation adherence probability pisol. The different curves show the effect of increasing the house-sharing factor H as labelled. (PDF) [file pone.0284805.s012.pdf]

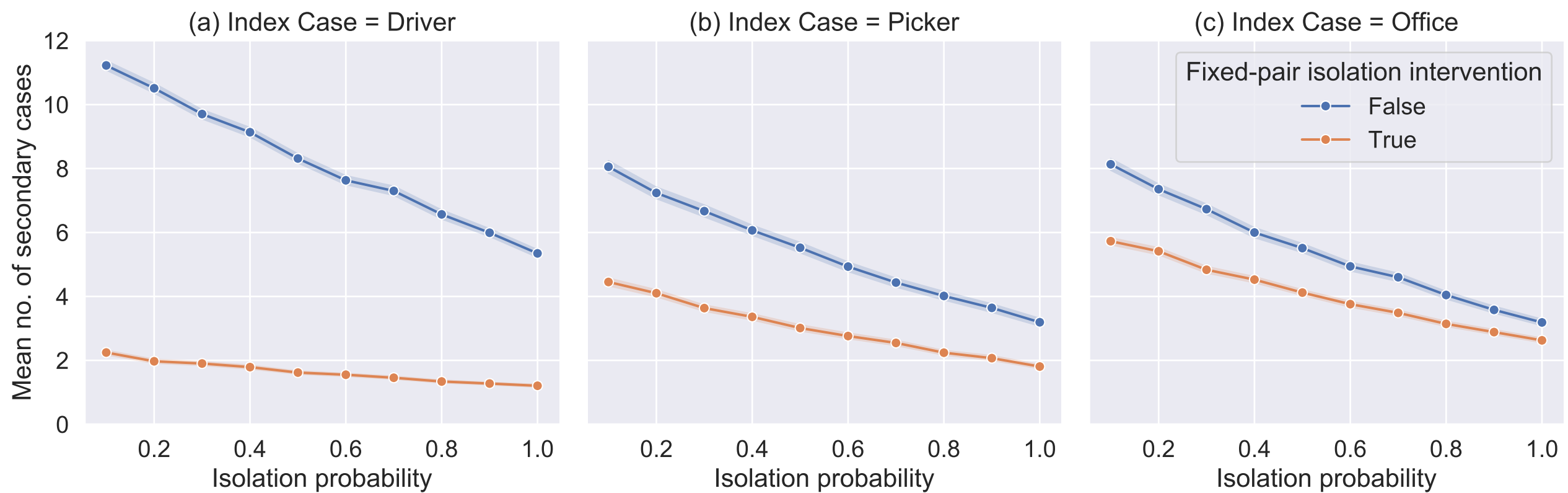

Supplement: S9 Fig — Dependence of mean number of simulated secondary cases from a single index case on the self-isolation adherence probability pisol. The different curves show the effect of adding a fixed-pairs isolation intervention. (PDF) [file pone.0284805.s013.pdf]

(a) Random pairs

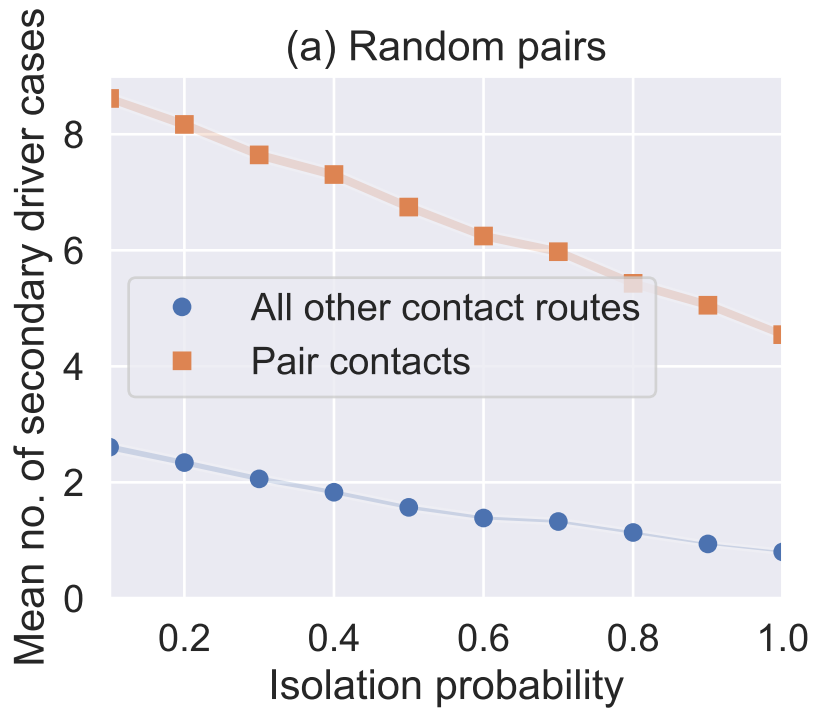

(b) Fixed pairs

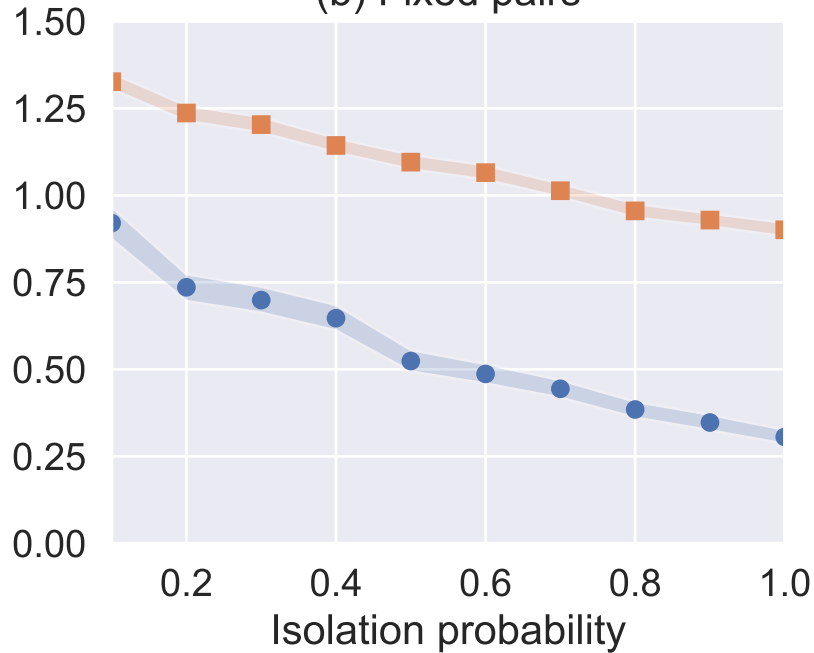

Supplement: S10 Fig — Mean number of infected drivers per simulation with a single driver index case plotted against symptomatic isolation probability pisol. The infections are broken down by those cased by close contact pair work, and all other contact routes. (a) The case with no fixed pairing intervention so pairs switch randomly each day. (b) The case with fixed pairings a pair isolation policy. Dots show the mean number of infections while shading shows 95% confidence in the mean calculated via bootstrapping methods. (PDF) [file pone.0284805.s014.pdf]

(a) Aerosol scale factor: 0.5

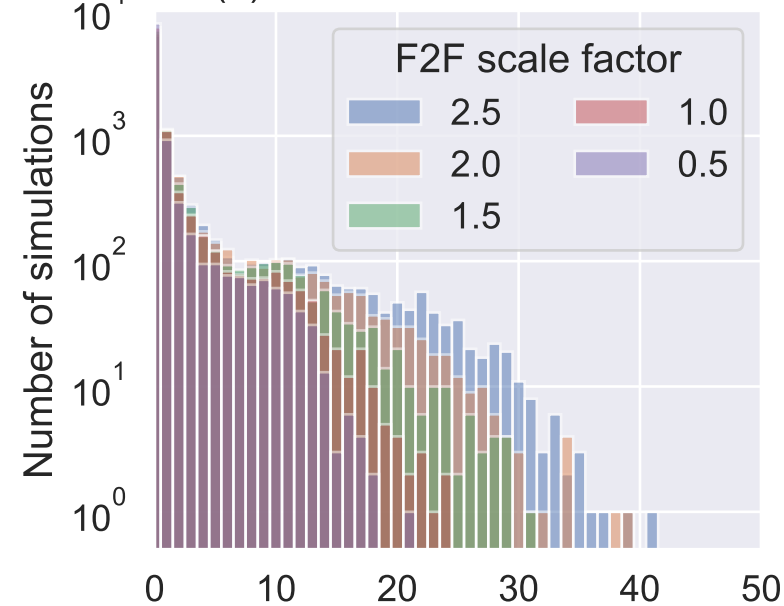

(b) Aerosol scale factor: 1.0

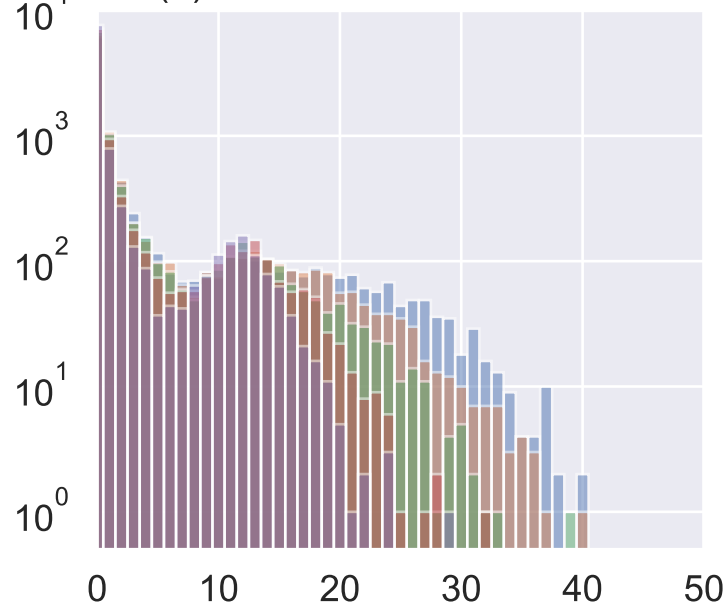

(c) Aerosol scale factor: 2.5

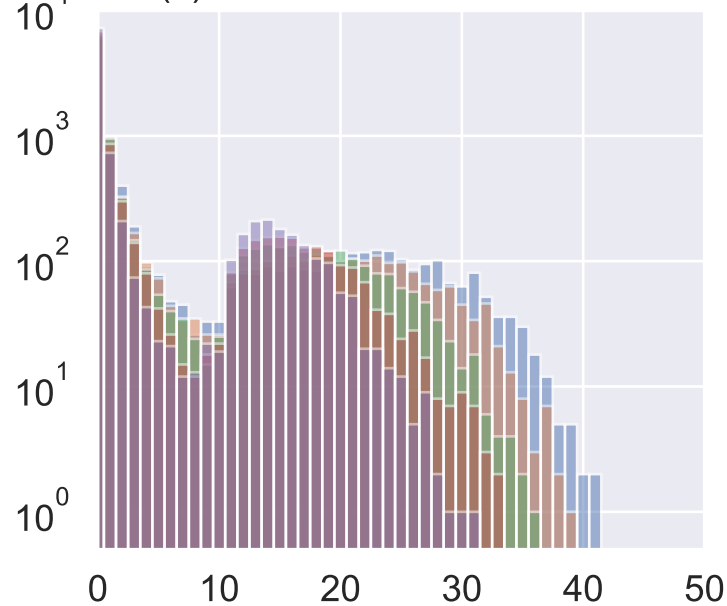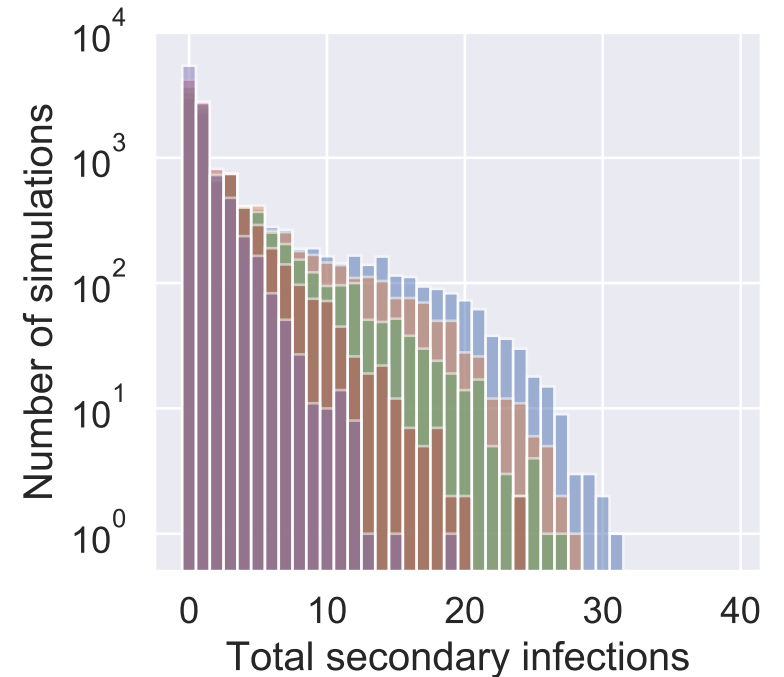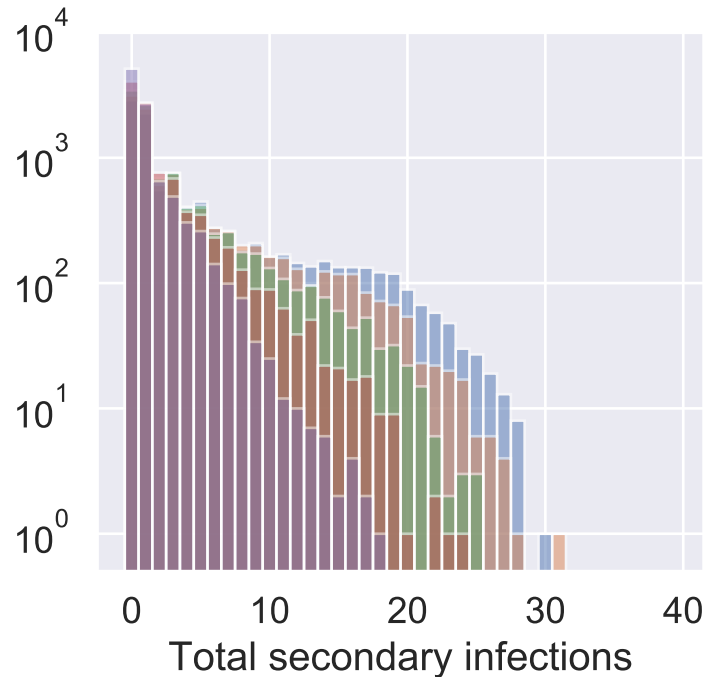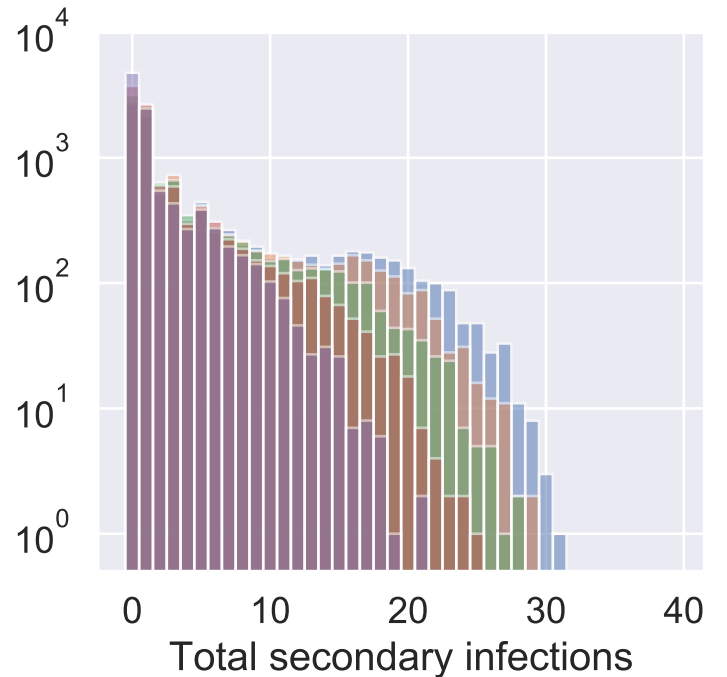

Supplement: S11 Fig — Histograms of secondary cases resulting from a single index case in the two work settings simulated for different rates of F2F and aerosol transmission. The top row shows the parcel work setting, while the bottom row is the large-item setting. For each set of simulations, the transmission rate for F2F contacts is multiplied by “F2F scale factor”, and the transmission rate for aerosol contacts is multiplied by “Aerosol scale factor”. Note that for the large-item workplace we assume that the fixed-pair isolation intervention is applied and in both cases pisol = 0.9. We also assume that the index case is selected randomly. (PDF) [file pone.0284805.s015.pdf]

(a) Parcel delivery setting

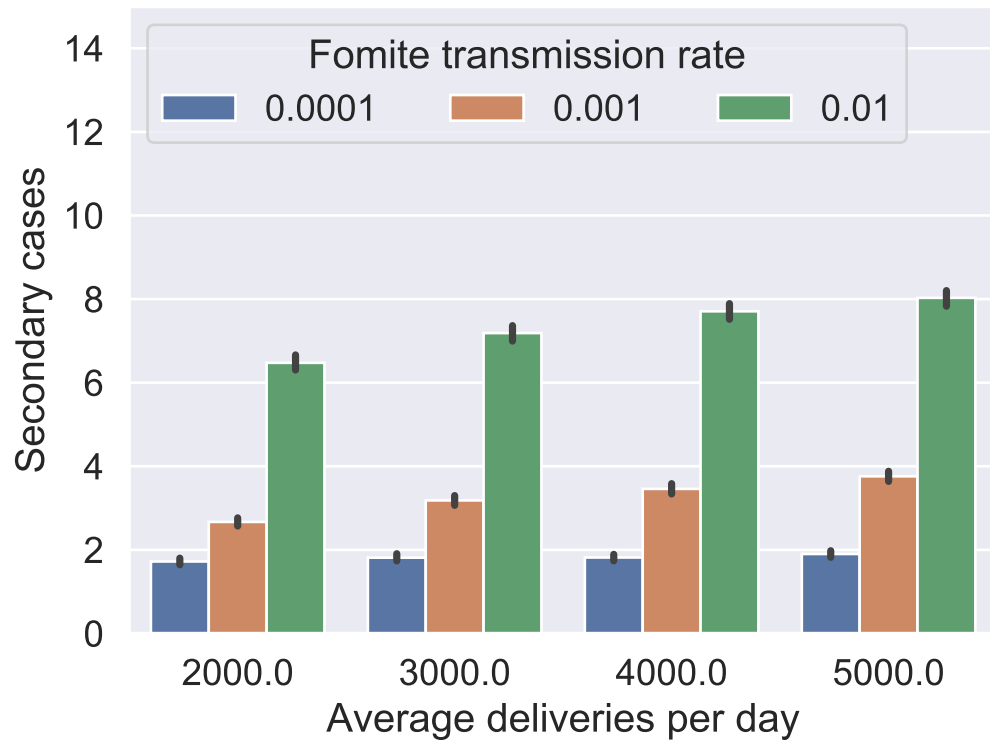

(b) Large-item delivery setting

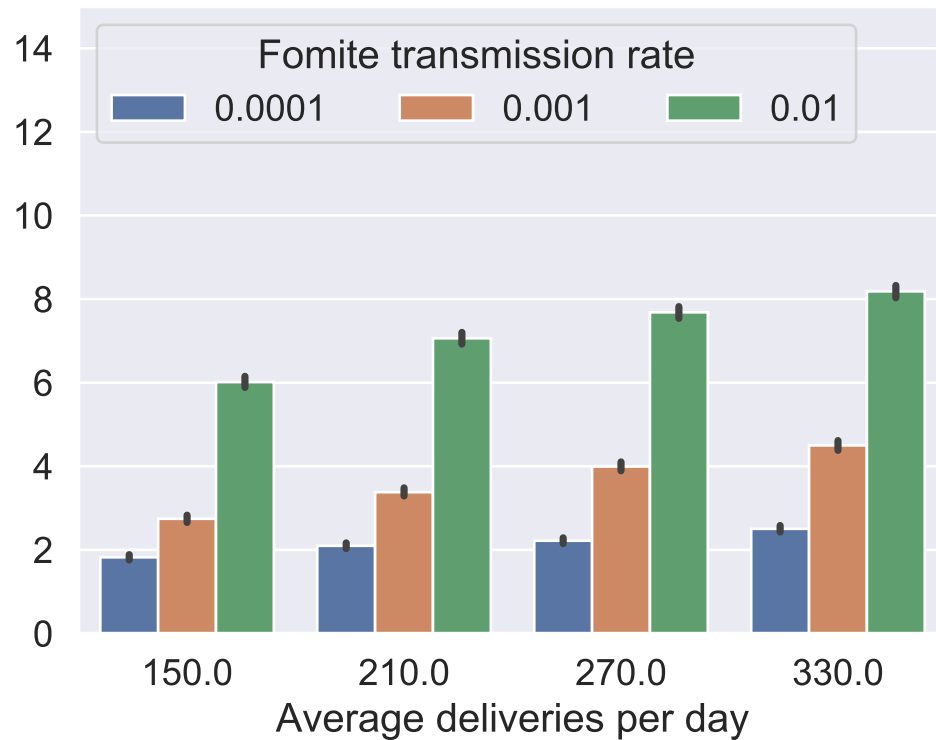

Supplement: S12 Fig — The mean number of secondary cases resulting from a single index case in the two workplace types plotted for 3 values of βFOM at varying levels of demand for deliveries (x-axis). Note that for the large-item workplace we assume that the fixed-pair isolation intervention is applied and in both cases pisol = 0.9. (PDF) [file pone.0284805.s016.pdf]

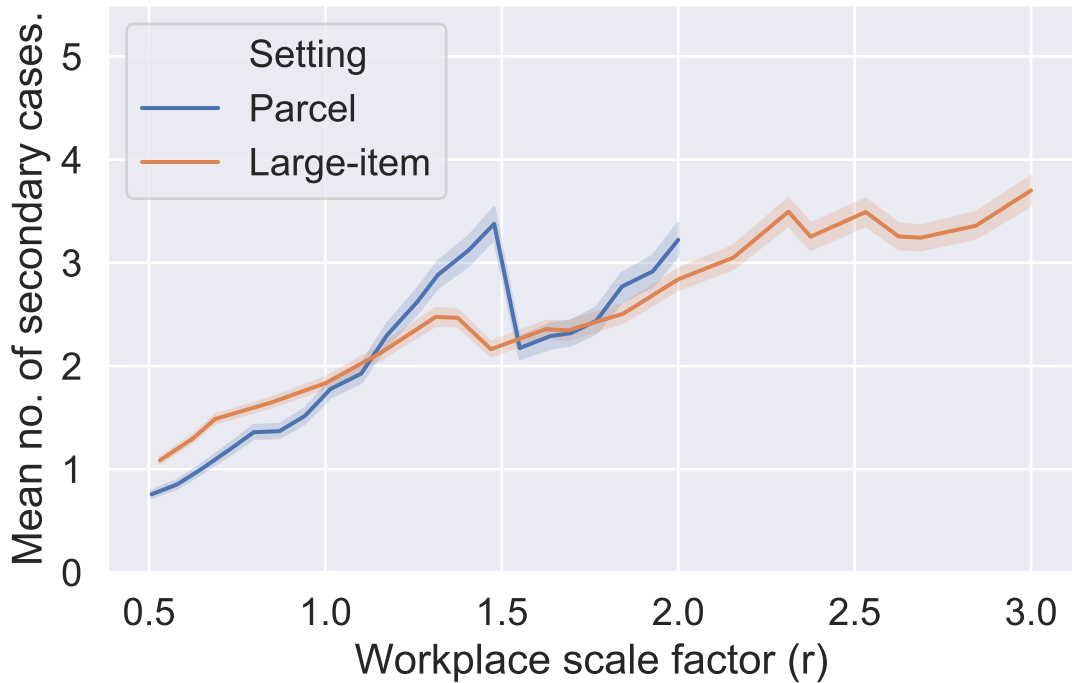

Supplement: S13 Fig — The mean number of secondary cases resulting from a point-source outbreak in the two workplace types plotted against workplace scale factor. Note that for the large-item workplace we assume that the fixed-pair isolation intervention is applied and in both cases pisol = 0.9. We assume the index case is selected at random. (PDF) [file pone.0284805.s017.pdf]

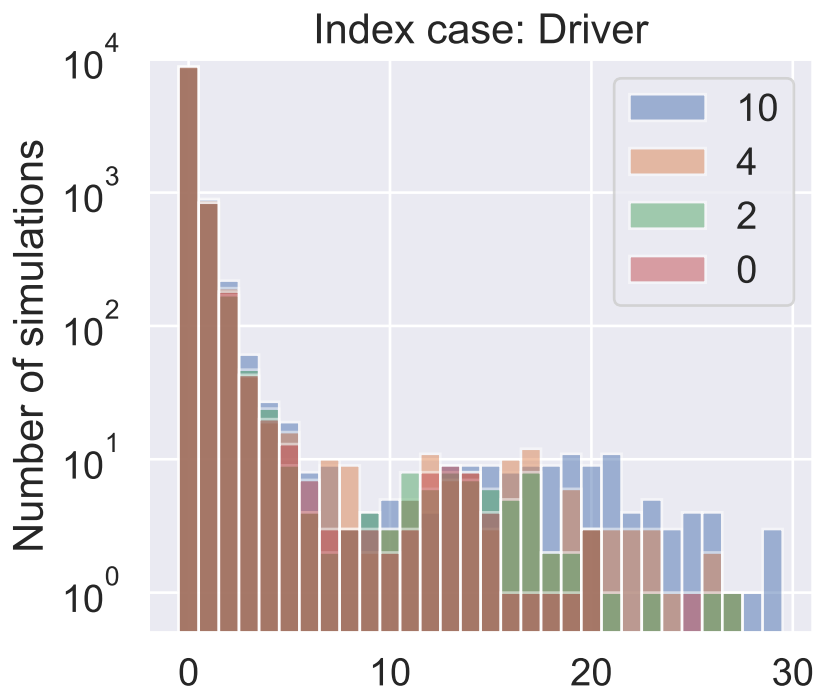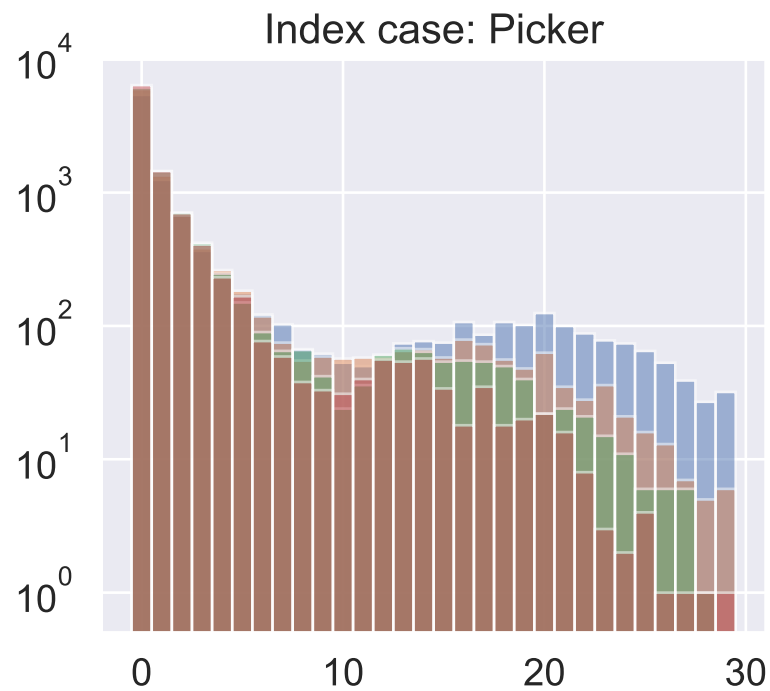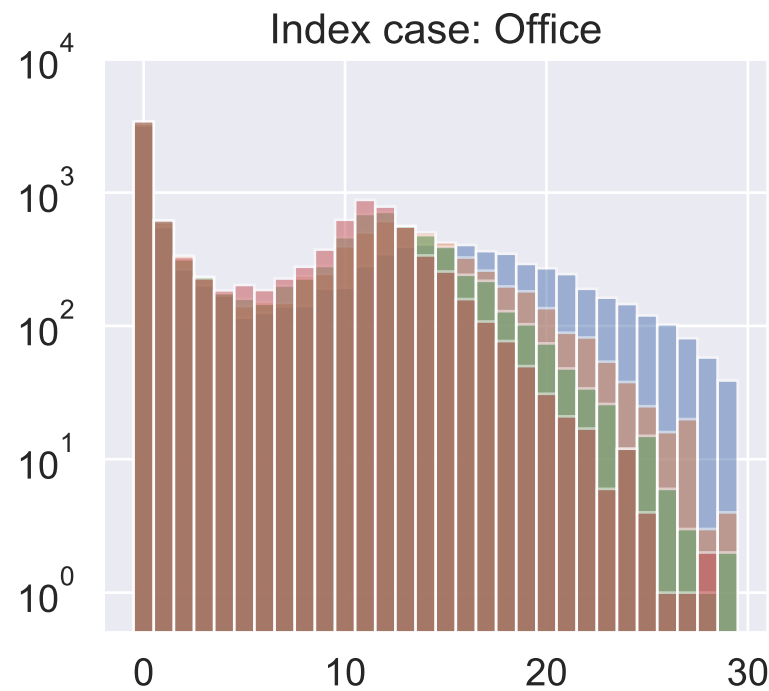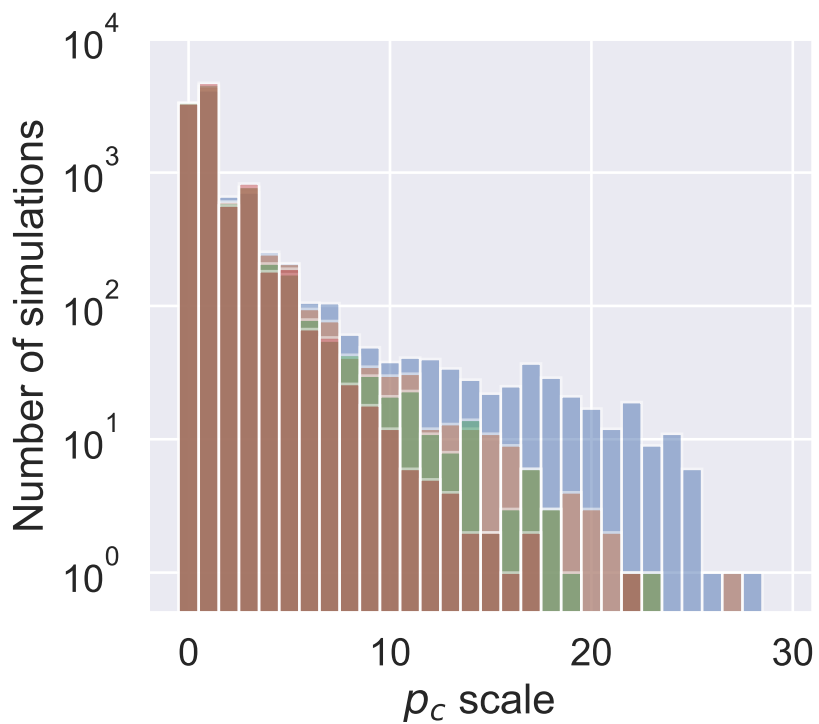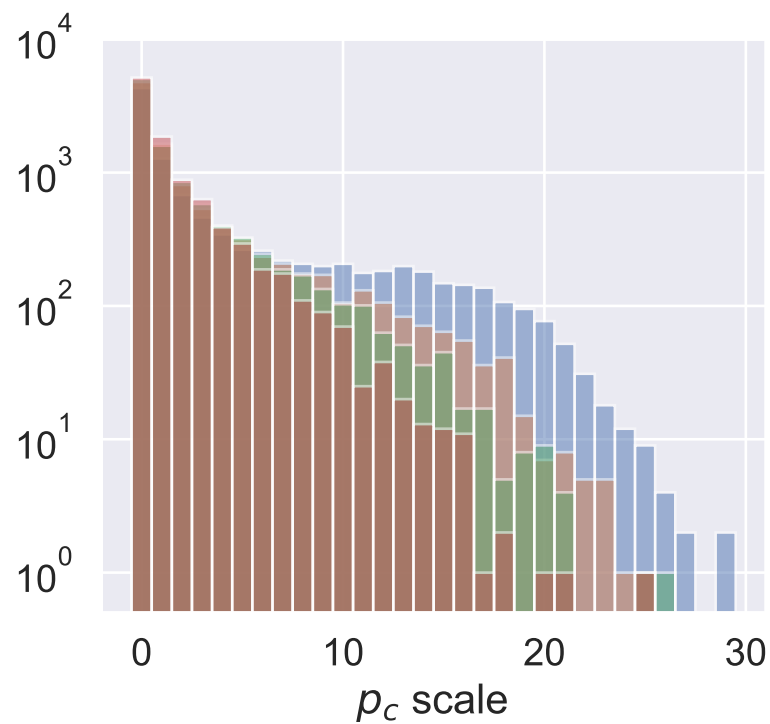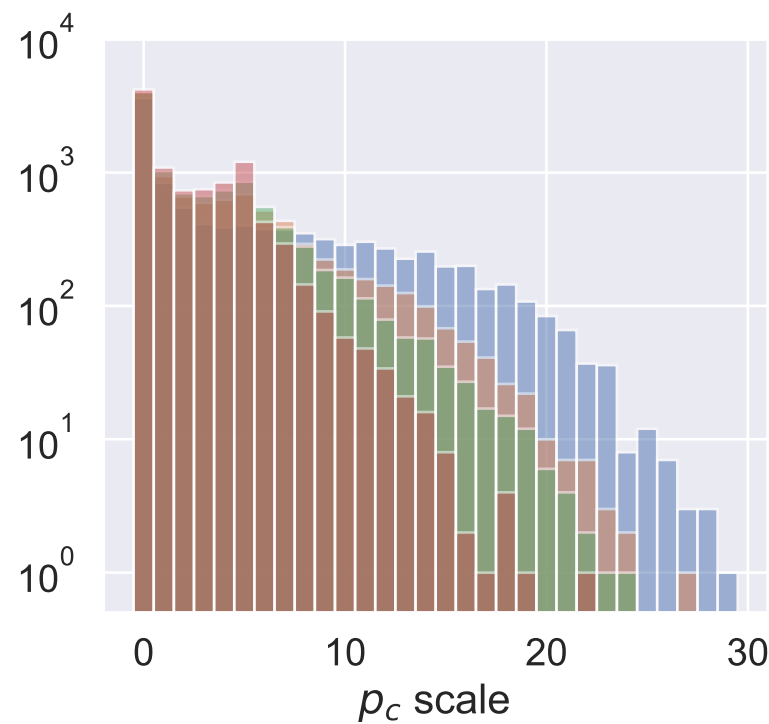

Supplement: S14 Fig — Histograms of the number of secondary cases resulting from a single index case in the two workplace types plotted for different scalings of pc(ND + NL + NO). The top row shows the parcel delivery setting, while the bottom row is large-item setting, and each column is for the index-case labelled. Note that for the large-item workplace we assume that the fixed-pair isolation intervention is applied and in both cases pisol = 0.9. Note also the logarithmic scale. (PDF) [file pone.0284805.s018.pdf]
