# Supplementary material for: Modelling the impact of non-pharmaceutical interventions on workplace transmission of SARS-CoV-2 in the home-delivery sector
Source: PLoS One. 2023 May 5;18(5):e0284805. doi: 10.1371/journal.pone.0284805 (PMC10162531; doi:10.1371/journal.pone.0284805)
Supplement: Attachment — Submitted filename: review_PONE-D-22-29161.pdf [file pone.0284805.s019.pdf]

1 Reviewer recommendation

Accept after minor changes.

2 Summary

The authors propose an agent-based model of SARS-COV-2 contagion in the workplace. The new model, which is parametrized using data and consultations, is used to study the effect of non-pharmaceutical interventions in the parcel delivery and logistics sector. Authors provide a detailed analysis of different interventions. Noteworthy, the model takes into account variability in the host viral load. The supplementary information provides complete details on all modeling decisions: data collection, parameter derivation, simulation algorithm, and baseline modeling. Appendix D: provides an analysis of sensitivity to assumptions and parameter choices. This is a good paper, and it should have broad applicability. However, this reviewer recommends the authors to address a few issues listed below before the manuscript is considered for publication.

3 Comments

Minor issues

Asynchronous vs synchronous updating. Please explain the rationale of synchronous updating in the number of infectious individuals in the simulation algorithm (Appendix B. Supplementary material) as opposed for example to Markov jump processes on networks. It is known that delay (due perhaps to synchronous updating) may induce oscillations in systems [3]. In the present case, it may happen that oscillations affect the time to extinction. On the other hand, it is known that synchronous and asynchronous updating yield different results in individual-based models [2, 1].

Behavior. Although the manuscript's findings have a lot of merits, accounting for behavior is a much-needed feature for a model of this kind. This is especially the case if a model is to be used to assist decision-making. Please discuss incorporating a stream of behavioral data into the present model, as suggested in the discussion.

Figure S13. Please explain more thoroughly the underlying mechanism behind the oscillation in the mean number of secondary cases as the workplace scale factor increases in Figure S13 *...the sharp reduction occurs when the number of teams is increased...* What is the purpose of showing that this oscillation occurs for a certain threshold?

References

[1] G. Caron-Lormier, R. W. Humphry, D. A. Bohan, C. Hawes, and P. Thorbek. Asynchronous and synchronous updating in individual-based models. *ecological modelling*, 212(3-4):522–527, 2008.

[2] B. A. Huberman and N. S. Glance. Evolutionary games and computer simulations. *Proceedings of the National Academy of Sciences*, 90(16):7716–7718, 1993.

[3] R. M. May. Stability and complexity in model ecosystems. In *Stability and Complexity in Model Ecosystems*. Princeton university press, 2019.
