# Supplementary material for: Modelling the impact of non-pharmaceutical interventions on workplace transmission of SARS-CoV-2 in the home-delivery sector
Source: PLoS One. 2023 May 5;18(5):e0284805. doi: 10.1371/journal.pone.0284805 (PMC10162531; doi:10.1371/journal.pone.0284805)
Supplement: Attachment — Submitted filename: response_to_refs.pdf [file pone.0284805.s020.pdf]

Dear editor and referees,

We would like to thank the referees for their reviews of our paper. We have provided a point-by-point response to their comments below. Excerpts from the new manuscript are highlighted in red. All of the editorial comments have also been addressed in the updated manuscript, as listed below.

Response to editorial comments

1. To match PLOS layout guidelines we have added a "Conclusion" section which consists of what was previously the concluding sentence of the Discussion section.
2. Updates to the data availability statement are contained in the cover letter.
3. See above
4. A lead author has been added for the consortium authorship, and the author list moved to the acknowledgements section
5. We have now added figure 4(c) so that the phrase 'data not shown' is no longer required.
6. The ethics statement has now been moved to the Methods section.
7. We have added captions for all the supplementary materials to the end of the document. To align with PLOS One layout guidelines, we have also separated the supplementary texts as submitted into the separate text, figure and table components now listed at the end of the document so that they can be referenced directly in the main document. We have also ensured that all supplementary material is cited in the main text.
8. The reference list has been reviewed

Reviewer 1

We thank the reviewer for their positive comments and constructive review, below is our response to the specific comments made.

1) *Conclusion:*
   Since the mathematical models developed in this study did not evaluate the transmission between workers and customers, I suggest removing this sentence "but that these posed minimal risk to customers", or to re-write "However, these could pose minimal risk to customers".

Our model does simulate contacts between workers and customers and the resulting customer infections. However, this had not been adequately highlighted in the manuscript. We have added the following paragraph to the results section (line 259) to highlight these results in a little more detail.

> Supplementary Fig S7(b) shows that this is also predicted to have a knock-on effect for customer infections, making them approximately as rare as in the parcel delivery workplace setting.

2) *Introduction:*
   Pag 4, paragraph 2, line 7: I suggest changing: "epidemiological data and data and …" for: "epidemiological data and …"

Corrected

3) *Pag 4, paragraph 3, line 4: Since there are antibody and antigen lateral flow tests, I suggest adding "antigen".*

We have added the qualifier “antigen” to all cases where we are discussing LFD antigen tests.

4) *Pag 10, paragraph 2: Household sharing is not a route of transmission. In this situation, the transmission occurs by the other three routes mentioned (F2F contact [droplets], indirect contact [air transmission], or fomite transmission). Household sharing is another condition such as car-share o room-share. For these reasons, I suggest removing this route from the sentence.*

We have removed household transmission from that sentence.

5) *I suggest including the definition of parcel the delivery workplace and the large-item delivery workplace (How many workers does each workplace has?).*

We have added the following paragraph at line 110, as well as introducing acronyms so that the parameters and results for two settings can be more easily distinguished:

> The model is parameterised to represent two archetypal delivery workplaces, a Small Parcel Delivery Depot (SPDD) and a Large-items Delivery Depot (LIDD). These represent depots that ship directly to customers. The SPDD is representative of a typical depot for (inter)national couriers shipping small packages that can be handled by a single person. The LIDD case represents a depot for logistics companies that specialise in items such as furniture and white goods, and may also offer installation/assembly of the products as part of delivery. As shown in table 1, the LIDD model has fewer staff, longer delivery times (as the deliveries tend to be more spatially separated), longer customer contact durations (because items tend to be delivered into the home and may be assembled/installed) and thus an order of magnitude fewer deliveries per day than the SPDD model.

6) *Results:*
Pag 17, paragraph 1: Review “… xx%...”

Now corrected to read 75% (and the previous figure which had read 75% should have read 80%). We have also added data to figure 4(c) to show this result.

7) *Figure 7. I suggest changing the title. For example, “Rate of Secondary cases and isolation days in a large-item delivery workplace over a 3-month period. Also, I suggest adding a footnote such as in Figure 6.*

Figure 8. I suggest changing the title. For example, “Rate of Secondary cases and isolation days by each intervention (rather than cumulatively). (a) Parcel delivery workplace. (b) large-item delivery workplace.”

Done

8) *Discussion:*
Pag 26, paragraph 3, line 3:
Since the mathematical models developed in this study did not evaluate the transmission between workers and customers, I suggest removing this part of the sentence “the risk of community”.

Given our response to comment 1) we have kept the reference to community transmission here, however we have reworded this slightly to make it clear that the interventions can directly reduce

staff to customer infection event but that we are not commenting on community transmission more generally.

Reviewer 2

We thank the reviewer for their positive comments and for the thorough review of our manuscript. Below we have responded to the specific concerns raised.

1) ***Asynchronous vs synchronous updating.*** *Please explain the rationale of synchronous updating in the number of infectious individuals in the simulation algorithm (Appendix B. Supplementary material) as opposed for example to Markov jump processes on networks. It is known that delay (due perhaps to synchronous updating) may induce oscillations in systems [3]. In the present case, it may happen that oscillations affect the time to extinction. On the other hand, it is known that synchronous and asynchronous updating yield different results in individual-based models [2, 1].*

This is an important point, and one that that we have now highlighted in the manuscript. There was no technical reason precluding the use of an asynchronous method (as the system could be discretised this way). The main reasons we used synchronised updating with a discretisation scale of 1 day can be summarised as follows:

- The shift and contact patterns (and hence the contact network) change from day-to-day. So, a discretisation of 1 day was a natural (and simple) choice
- Newly infected individuals have negligible infectiousness for the first day, so the chance of them infecting another colleague on the same day, which would introduce error compared to an asynchronous update, are very rare.
- We aimed for the model to be transparent and easy to generalise/adapt to other settings and this appeared to be the best framework for that while remaining computationally efficient and avoiding being restricted to certain system classes (e.g. Markovian).

We have added the following text to the discussion to highlight this:

> The simulations employ an individual-based network model approach with daily contact networks randomly generated using the parameterisations in table 2. The algorithm updates contacts and infection events at discrete intervals of one day. This was chosen as the most natural option because the contact network changes from day-to-day. Additionally, the data collected to parameterise the model (including viral load data) is all defined at the scale of 1 measurement per day. However, this ``synchronous’’ updating does introduce some error into the dynamics of the simulated epidemiology. It is known that in generic individual-based models synchronous updating can cause spurious oscillations in the dynamics compared to asynchronous methods such as a Gillespie algorithm or Markov chain model [35]. Here a synchronous method was employed to make the model more transparent and generalisable (e.g. to non-Markovian processes), and to avoid the complexity of specifying the timings of shift and contact patterns over the course of a single day. This is similar to other recent network or IB epidemic models [5,7,9]. We justify this by reasoning that the error introduced is likely to be insignificant for transmission of SARS-CoV-2 as a newly infected individual is effectively non-infectious for the first day. Therefore, events where one worker is infected and then infects a co-worker within the same shift, which are missed by the synchronous update model, are vanishingly rare. Thus, there is no mechanism to trigger oscillations in this system at the timescale of the discretisation. The algorithm is outlined in detail in Supplementary Text S2.

2) ***Behavior***. *Although the manuscript's findings have a lot of merits, accounting for behavior is a much-needed feature for a model of this kind. This is especially the case if a model is to be used to assist decision-making. Please discuss incorporating a stream of behavioral data into the present model, as suggested in the discussion.*

We have added the following text to the discussion to highlight ways that behavioural data can be incorporated into the model.

On improving models of contact behaviour:

> This could be improved if data were available from e.g. wireless proximity sensors, as have been used in other studies to reconstruct social contact networks [41,42], including in workplaces [43]. These provide much more high-fidelity data but when data is collected during an epidemic or while restrictions are in place, these devices can themselves affect behaviour and encourage greater distancing/policy adherence with a number of devices deployed during the pandemic actively designed to have this effect [44,45]. Therefore, empirical contact networks in the absence and presence of restrictions are difficult to ascertain. Behaviour around social distancing is difficult to simulate, so contact networks based on proximity monitors would be a significant improvement, especially if they were deployed while measures were introduced.

We have also expanded the discussion around improving models of testing behaviour and adherence to other work policies:

> Second, we do not model the complex relationship between interventions and behaviour. It is possible that as more interventions are introduced, adherence with other interventions wanes so the expected impact of combined interventions may not be as high as predicted. This behavioural change is difficult to predict, and so would need to be monitored by companies to gauge whether interventions are working as expected. Furthermore, even with high adherence there is no guarantee that people will use the test as intended. For example, people may be inclined to test more regularly when feeling `run down' or `paucisymptomatic', i.e. exhibiting very mild COVID-19 symptoms, whereas in the absence of testing they may have simply isolated from work. In this case, much of the benefit of testing can be lost [53] because asymptomatic carriers will be less likely to be detected while symptomatic carriers who would have otherwise isolated may be given a false negative and choose not to. For this reason, in some sectors, mandatory regular testing (i.e. carried out by trained swabbers at the workplace) may be the preferred option, because with the adherence rates assumed in this paper, one mandatory test per week has a similar impact to two voluntary ones (see figures 3 and 4). To address this shortcoming of the model, surveys of staff or test reporting rates in relevant sectors where regular testing has been deployed may inform changes. In particular, data around when and how tests were being used would be useful (as well as rates of symptomatic isolations). Survey information regarding contact frequency with other employees while off-work or in isolation would also inform the model assumptions around the effectiveness of isolation measures in reducing contacts. Finally, data from workplaces that monitor adherence to other intervention policies (such as mask-wearing) could inform the adherence rates simulated here. However with all behavioural and survey data, there is the risk of reporting bias and behavioural changes in response to observation.

3) ***Figure S13.*** *Please explain more thoroughly the underlying mechanism behind the oscillation in the mean number of secondary cases as the workplace scale factor increases in Figure S13*

...the sharp reduction occurs when the number of teams is increased... What is the purpose of showing that this oscillation occurs for a certain threshold?

Figure S13 is intended to show the range of outcomes for parcel and large-item delivery settings across the range of workplace sizes we predict from the delivery data we have processed. We reasoned that as the workplace size increases the number of workplace teams will also increase (to keep the team size approximately consistent). However, these are discrete thresholds, which are triggered at the points where you see a sharp decrease in infections. This shows that some of the effects of changing workplace size are simply due to office sizes increasing (the effect shown in figure S6), but that this does not completely account for all of the increase in infections with size.

We have clarified the supplementary text accordingly. In particular we have removed the discussion about outbreak threshold (this seems to have mistakenly been left in from a previous version, where the figure showed outbreak probability rather than secondary cases). To remove this artefact of choice of outbreak threshold we show secondary cases instead. The new text reads

> Figure S13 shows the results of point-source outbreak simulations in the two settings across the range of feasible workplace size scalings. We see that the number of secondary cases resulting from the outbreak increases with workplace size, although this is, in part, explained by the increase in office cohort size (the sharp reduction occurs when the number of teams is increased). This is because the number of teams are chosen relative to the number of employees (to keep the number per team as consistent as possible). However, these changes in team numbers occur at discrete thresholds, and as shown in figure S6. This shows that some of the effects of changing workplace size are simply due to office sizes increasing (the effect shown in figure S6), but that this does not completely account for all of the increase in infections with size.
